# Supplementary material for: Pathway-guided monitoring of the disease course in bladder cancer with longitudinal urine proteomics
Source: Commun Med (Lond). 2023 Jan 16;3:8. doi: 10.1038/s43856-023-00238-4 (PMC9842762; doi:10.1038/s43856-023-00238-4)
Supplement: Supplementary file 1 — Description of Additional Supplementary Files [file 43856_2023_238_MOESM1_ESM.pdf]

Description of Additional Supplementary Files

File Name: Supplementary Data 1

Description: Proteins that were differentially expressed between the BC-T1 group with recurrence and/or progression and the BC-T1group with no recurrence.

File Name: Supplementary Data 2

Description: Source data for the Figures. 1, 2, 3, 4 and Supplementary Figures 2, 3, 4, 5 presented in the manuscript.
