# Supplementary material for: Pathway-guided monitoring of the disease course in bladder cancer with longitudinal urine proteomics
Source: Commun Med (Lond). 2023 Jan 16;3:8. doi: 10.1038/s43856-023-00238-4 (PMC9842762; doi:10.1038/s43856-023-00238-4)
Supplement: Supplementary file 2 — Supplementary Material [file 43856_2023_238_MOESM2_ESM.pdf]

Supplementary Material

Pathway-guided monitoring of the disease course in bladder cancer with longitudinal urine proteomics

Luís Botelho Carvalho[a,b], José Luis Capelo[a,b,], Carlos Lodeiro[a,b], Rajiv Dhir[c], Luis Campos Pinheiro[d,e], Hugo López-Fernández [f,g], Gonçalo Martins [a,b], Mariana Medeiros[d,e], Fernando Díaz [h] & Hugo Miguel Santos[a,b,c,*]

[a] BIOSCOPE Research Group, LAQV-REQUIMTE, Department of Chemistry, NOVA School of Science and Technology, Universidade NOVA de Lisboa 2829-516, Caparica, Portugal.

[b] PROTEOMASS Scientific Society, Madan Parque, Rua dos Inventores, 2825-182 Caparica, Portugal.

[c] Department of Pathology, University of Pittsburgh Medical Center, Pittsburgh, PA, United States.

[d] Urology Department, Central Lisbon Hospital Center, Lisbon, Portugal.

[e]NOVA Medical School. NOVA University of Lisbon, Lisbon, Portugal.

[f] CINBIO, Universidade de Vigo, Department of Computer Science, ESEI-Escuela Superior de Ingeniería Informática, 32004 Ourense, Spain.

[g] SING Research Group, Galicia Sur Health Research Institute (IIS Galicia Sur), SERGAS-UVIGO, 36213 Vigo, Spain.

[h] Universidad de Valladolid, Department of Computer Science, Escuela de Ingeniería Informática, 40005 Segovia, Spain.

[*]Corresponding author: H.M.S. hmsantos@fct.unl.pt

Supplementary Methods

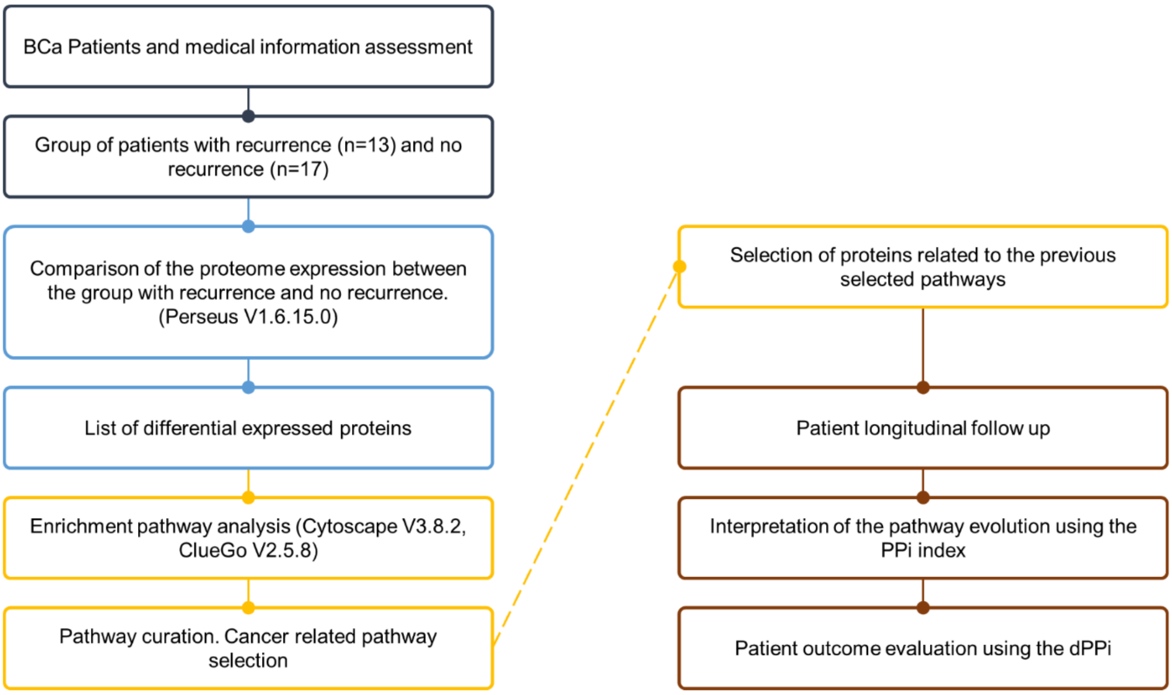

Supplementary Fig. 1. Bioinformatics analysis workflow. List of differentially expressed proteins was obtained with a Student´s t test (FDR 0.05 and S_0 of 0.1). Reactome and biological process GO terms for the enrichment pathway analysis were used as ontologies databases. The curation of pathways was carried out based on the enrichment pathways analysis from ClueGo. Pathways with 60% or more up-regulated proteins and lower p-values were selected. Furthermore, those pathways were crosscheck with a bibliography to select the ones correlated with Hanahan and Weinberg biological hallmarks of cancer [1,2] as shown in as shown in Supplementary Table 1.

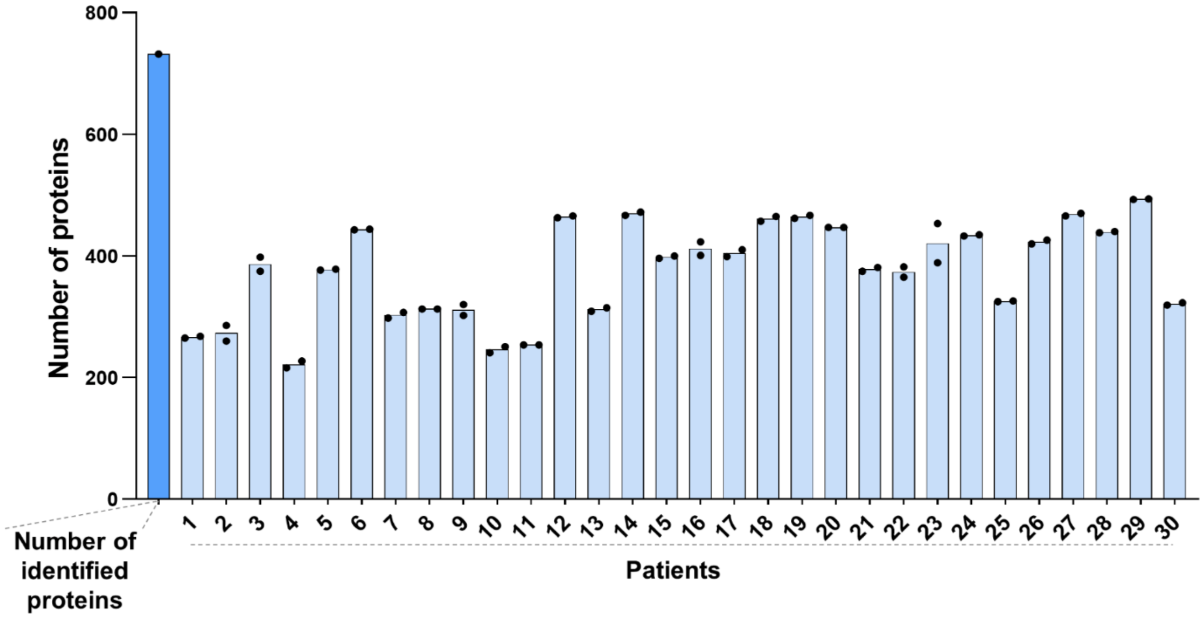

Supplementary Fig. 2. Proteomics data summary with the number of identified proteins for each patient. Bars represent the average of two technical replicates.

Supplementary Discussion

Patient IV was diagnosed with T1 stage bladder cancer (BC) and was submitted to the standard TURBT procedure. One reference urine sample was taken at the disease onset before the medical intervention, as shown in Fig. SM3A, where a comprehensive clinical outcome timeline for 25 months, including the sampling time points, is shown. Control urine was taken ten days after surgical intervention, and the lower values for all the pathways are reflected in a dPPi value of -80, indicating an amelioration period. However, 25 months later, all the pathways presented higher levels, with a dPPi value of -56. The increase in the MAPK pathway and the increment in the levels of the APOA4 and APOE biomarkers indicate a worsening of the disease course. Also, the cluster shows that the sampling point three patient's proteomics profile (25 months) is similar to the one present in sampling point one, Fig. SM3F. Under our request, the patient is waiting for cystoscopy revision.

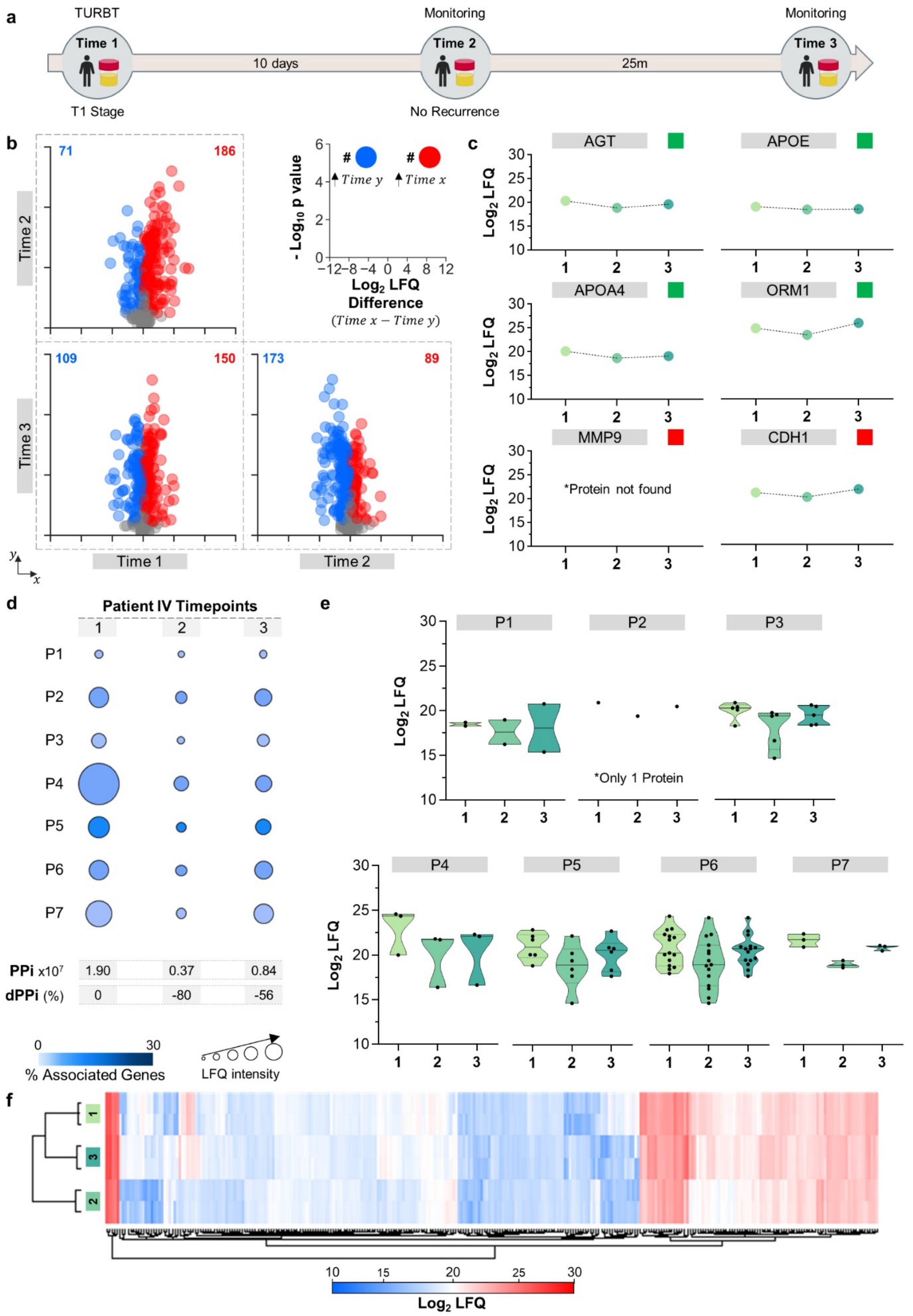

Supplementary Fig. 3. Longitudinal analysis of the urinary proteome of patient IV. a Timeline of disease course in Patient IV showing the intervals in months (m) between medical interventions and

urine sampling (time 1 to time 3). TURBT: transurethral resection of bladder tumour. **b** Volcano plot matrix showing proteome changes in the urine collected at timepoints 1-3. The dots represent the proteins that showed statistically significant increases (red), decreases (blue) or nonstatistically significant changes (grey) according to Student´s *t* test (FDR 0.05 and S0 of 0.1). **c** Variation in the known protein biomarkers for bladder cancer: angiotensinogen (AGT)[3], apolipoprotein E (APOE)[4], matrix metalloproteinase-9 (MMP9)[5], apolipoprotein A-IV (APOA4)[6], alpha-1-acid glycoprotein 1 (ORM1)[7] and cadherin-1 (CDH1)[8,9]. The green and red squares indicate whether the biomarker variation in the urine of Patient V matched or not, respectively, with trends reported in the literature (considering only the first two sampling points). Dots represent the average of two biological samples with two technical replicates each. **d** Personal pathway index (PPi) at each time point and the estimated differential PPI (dPPi) were calculated as explained in the text. P1: interleukin-12-mediated signalling pathway; P2: endodermal cell differentiation; P3, proteoglycan binding; P4: peroxidase activity; P5: complement cascade; P6: humoral immune response; P7: oncogenic MAPK signalling. **e** Distribution and density variation protein LFQ values (including two biological replicates) at each sampling point for each pathway assessed. Continuous bar in the middle represents the median. The thin discontinuous line represents the quartile lines. **f** Hierarchical clustering of the three urinary proteomes of Patient IV. The protein LFQ values were used to perform the cluster analysis (with average linkage, no constraint, preprocessing with k-means and Euclidean distance).

Patient V followed our standard procedure. As he presented for a potential BC, one urine was taken, and a cystoscopy was carried out. Because T1 stage BC was diagnosed, a TURBT was performed. The comprehensive timeline analysis of patient V is shown in Fig SM5A. The evolution of the urine proteome reveals that this patient is not responding to medical surgery and treatment. As a matter of fact, after one month, the levels of the hallmark pathways were virtually the same. Then, a new cystoscopy was done at our request, and as a recurrence was detected, a new TURBT was done. Seven months later, the dPPi remains almost the same, the MAPK pathway presents higher when compared with the onset, and the single biomarkers APOA4 and APOE shows an increment. Taking it all together, we concluded that a new cystoscopy was needed. Afterwards, the patient was diagnosed with a Nonneoplastic bladder mass that was removed.

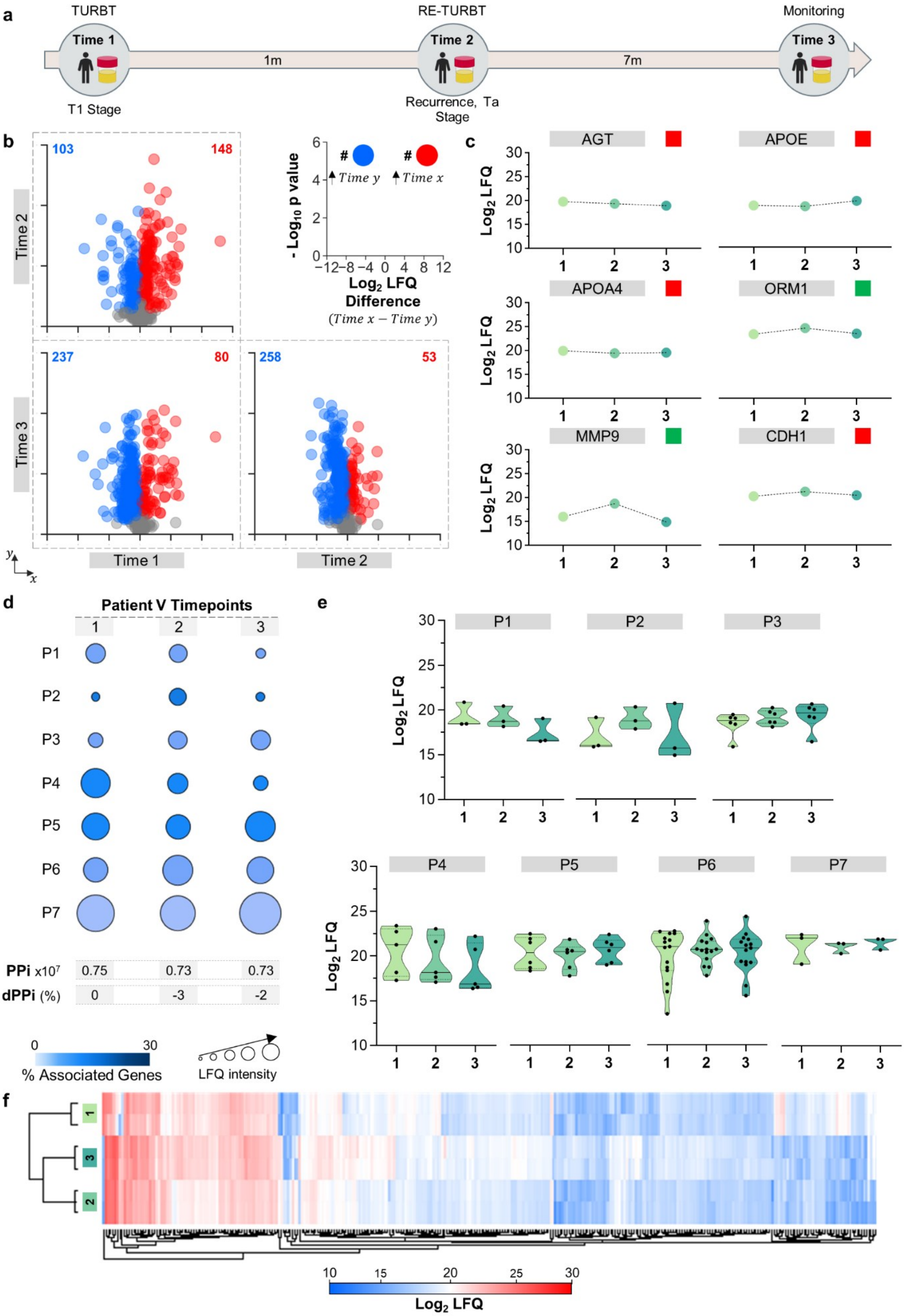
a
TURBT
Time 1
T1 Stage
1m
RE-TURBT
Time 2
Recurrence, Ta Stage
7m
Monitoring
Time 3
b
103
148
Time 2
237
80
258
53
Time 3
Time 1
Time 2
- Log10 p value
Time y
Time x
Log2 LFQ Difference
(Time x − Time y)
c
AGT
APOE
APOA4
ORM1
MMP9
CDH1
Log2 LFQ
d
Patient V Timepoints
P1
P2
P3
P4
P5
P6
P7
PPi x10^7 0.75 0.73 0.73
dPPi (%) 0 -3 -2
% Associated Genes
LFQ intensity
e
P1
P2
P3
P4
P5
P6
P7
Log2 LFQ
f
Log2 LFQ

Supplementary Fig. 4. Longitudinal analysis of the urinary proteome of patient V. a Timeline of disease course in Patient V showing the intervals in months (m) between medical interventions and urine sampling (time 1 to time 4). TURBT: transurethral resection of bladder tumour. **b** Volcano plot matrix showing proteome changes in the urine collected at timepoints 1-3. The dots represent the proteins that showed statistically significant increases (red), decreases (blue) or nonstatistically significant changes (grey) according to the Student´s *t* test (FDR 0.05 and S0 of 0.1). **c** Variation in the known protein biomarkers for bladder cancer: angiotensinogen (AGT)[3], apolipoprotein E (APOE)[4], matrix metalloproteinase-9 (MMP9)[5], apolipoprotein A-IV (APOA4)[6], alpha-1-acid glycoprotein 1 (ORM1)[7] and cadherin-1 (CDH1)[8,9]. The green and red squares indicate whether the biomarker variation in the urine of Patient V matched or not, respectively, with trends reported in the literature (considering only the first two sampling points). Dots represent the average of two biological samples with two technical replicates each. **d** Personal pathway index (PPi) at each time point and the estimated differential PPI (dPPi) were calculated as explained in the text. P1: interleukin-12-mediated signalling pathway; P2: endodermal cell differentiation; P3, proteoglycan binding; P4: peroxidase activity; P5: complement cascade; P6: humoral immune response; P7: oncogenic MAPK signalling. **e** Distribution and density variation protein LFQ values (including two biological replicates) at each sampling point for each pathway assessed. Continuous bar in the middle represents the median. The thin discontinuous line represents the quartile lines. **f** Hierarchical clustering of the three urinary proteomes of Patient V. The protein LFQ values were used to perform the cluster analysis (with average linkage, no constraint, preprocessing with k-means and Euclidean distance).

Patient VI was diagnosed as T1 BC after cystoscopy and resection. 25 months later a urinary analysis revealed a dPPi level 41% lower than the one in the onset, with all the pathway hallmarks presenting lower values. In addition, the single markers MMP9 and APOA4 showed lower values. Our diagnosis was a good evolution of the patient, which the physicians confirmed via cystoscopy. However, eight months later, routine urine surveillance revealed an increment of the dPPi from -41% to -7%, with a general increment in all the pathway hallmarks. An analysis done using the cells present in the urine revealed no recurrence. However, as the single markers MMP9, APOA4 and APOE were found augmented, we have requested new urine to follow up the course of this patient before recommending a cystoscopy.

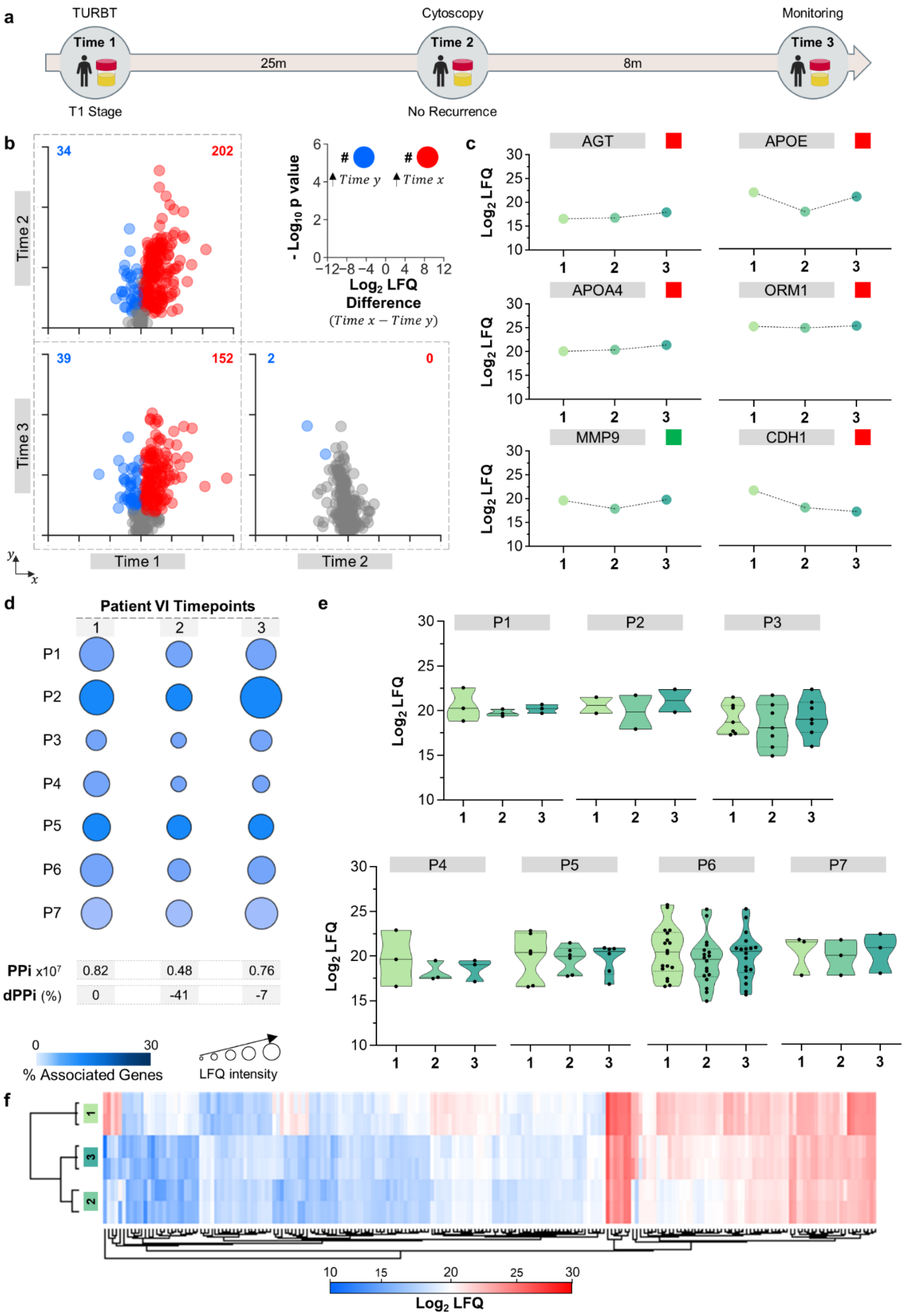

Supplementary Fig. 5. Longitudinal analysis of the urinary proteome of patient VI. a Timeline of disease course in Patient VI showing the intervals in months (m) between medical interventions and

urine sampling (time 1 to time 3). TURBT: transurethral resection of bladder tumour. **b** Volcano plot matrix showing proteome changes in urine collected at timepoints 1-3. The dots represent the proteins that showed statistically significant increases (red), decreases (blue) or nonstatistically significant changes (grey) according to Student´s *t* test (FDR 0.05 and S0 of 0.1). **c** Variation in the known protein biomarkers for bladder cancer: angiotensinogen (AGT)[3], apolipoprotein E (APOE)[4], matrix metalloproteinase-9 (MMP9)[5], apolipoprotein A-IV (APOA4)[6], alpha-1-acid glycoprotein 1 (ORM1)[7] and cadherin-1 (CDH1)[8,9]. The green and red squares indicate whether the biomarker variation in the urine of Patient V matched or not, respectively, with trends reported in the literature (considering only the first two sampling points). Dots represent the average of two biological samples with two technical replicates each. **d** Personal pathway index (PPi) at each time point and the estimated differential PPI (dPPi) were calculated as explained in the text. P1: interleukin-12-mediated signalling pathway; P2: endodermal cell differentiation; P3, proteoglycan binding; P4: peroxidase activity; P5: complement cascade; P6: humoral immune response; P7: oncogenic MAPK signalling. **e** Distribution and density variation protein LFQ values (including two biological replicates) at each sampling point for each pathway assessed. Continuous bar in the middle represents the median. The thin discontinuous line represents the quartile lines. **f** Hierarchical clustering of the three urinary proteomes of Patient VI. The protein LFQ values were used to perform the cluster analysis (with average linkage, no constraint, preprocessing with k-means and Euclidean distance).

Supplementary References

1. Hanahan, D. & Weinberg, R. A. The hallmarks of cancer. *Cell* vol. 100 57–70 (2000).

2. Hanahan, D. & Weinberg, R. A. Hallmarks of cancer: The next generation. *Cell* vol. 144 646–674 (2011).

3. Yoshida, T. *et al.* Prognostic impact of renin-angiotensin inhibitors in patients with bladder cancer undergoing radical cystectomy. *Ann. Surg. Oncol.* **24**, 823–831 (2017).

4. Ren, L. *et al.* Apolipoproteins and cancer. *Cancer Med.* **8**, 7032–7043 (2019).

5. Zeng, F. C., Cen, S., Tang, Z. Y. & Kang, X. L. Elevated matrix metalloproteinase-9 expression may contribute to the pathogenesis of bladder cancer. *Oncol. Lett.* **11**, 2213–2222 (2016).

6. Soukup, V. *et al.* The significance of calprotectin, CD147, APOA4 and DJ-1 in non-invasive detection of urinary bladder carcinoma. *Neoplasma* **66**, 1019–1023 (2019).

7. Li, F. *et al.* The increased excretion of urinary orosomucoid 1 as a useful biomarker for bladder cancer. *Am. J. Cancer Res.* **6**, 331–40 (2016).

8. Gurhan Balci, M. & Tayfur, M. *Loss of E-cadherin expression in recurrent non-invasive urothelial carcinoma of the bladder*. *Int J Clin Exp Pathol* vol. 11 www.ijcep.com/ (2018).

9. Pećina-Šlaus, N. Tumor suppressor gene E-cadherin and its role in normal and malignant cells. *Cancer Cell International* vol. 3 17 (2003).
