# Supplementary material for: Pathway-guided monitoring of the disease course in bladder cancer with longitudinal urine proteomics
Source: Commun Med (Lond). 2023 Jan 16;3:8. doi: 10.1038/s43856-023-00238-4 (PMC9842762; doi:10.1038/s43856-023-00238-4)
Supplement: Supplementary file 6 — Reporting Summary [file 43856_2023_238_MOESM6_ESM.pdf]

nature portfolio

Corresponding author(s): Hugo M. Santos

Last updated by author(s): Nov 28, 2022

Reporting Summary

Nature Portfolio wishes to improve the reproducibility of the work that we publish. This form provides structure for consistency and transparency in reporting. For further information on Nature Portfolio policies, see our Editorial Policies and the Editorial Policy Checklist.

Statistics

For all statistical analyses, confirm that the following items are present in the figure legend, table legend, main text, or Methods section.

n/a	Confirmed	
☐	☒	The exact sample size (*n*) for each experimental group/condition, given as a discrete number and unit of measurement
☐	☒	A statement on whether measurements were taken from distinct samples or whether the same sample was measured repeatedly
☐	☒	The statistical test(s) used AND whether they are one- or two-sided<br>*Only common tests should be described solely by name; describe more complex techniques in the Methods section.*
☐	☒	A description of all covariates tested
☐	☒	A description of any assumptions or corrections, such as tests of normality and adjustment for multiple comparisons
☐	☒	A full description of the statistical parameters including central tendency (e.g. means) or other basic estimates (e.g. regression coefficient) AND variation (e.g. standard deviation) or associated estimates of uncertainty (e.g. confidence intervals)
☐	☒	For null hypothesis testing, the test statistic (e.g. *F*, *t*, *r*) with confidence intervals, effect sizes, degrees of freedom and *P* value noted<br>*Give P values as exact values whenever suitable.*
☒	☐	For Bayesian analysis, information on the choice of priors and Markov chain Monte Carlo settings
☐	☒	For hierarchical and complex designs, identification of the appropriate level for tests and full reporting of outcomes
☒	☐	Estimates of effect sizes (e.g. Cohen's *d*, Pearson's *r*), indicating how they were calculated

Our web collection on statistics for biologists contains articles on many of the points above.

Software and code

Policy information about availability of computer code

Data collection	Bruker Compass otof Series 3.4; HyStar v3.2
Data analysis	MaxQuant software V2.0.3.0, Perseus v1.6.15.0, Cytoscape V3.8.2, ClueGo V2.5.8 and GraphPad Prism 8.2.1.

For manuscripts utilizing custom algorithms or software that are central to the research but not yet described in published literature, software must be made available to editors and reviewers. We strongly encourage code deposition in a community repository (e.g. GitHub). See the Nature Portfolio guidelines for submitting code & software for further information.

Data

Policy information about availability of data

All manuscripts must include a data availability statement. This statement should provide the following information, where applicable:

- Accession codes, unique identifiers, or web links for publicly available datasets
- A description of any restrictions on data availability
- For clinical datasets or third party data, please ensure that the statement adheres to our policy

The mass spectrometry proteomics data that support the findings of this study have been deposited in ProteomeXchange Consortium via the PRIDE partner with the PXD025139 accession codes. Source data for the Figures. 1, 2, 3, 4 and Supplementary Figures 2, 3, 4, 5 presented in the manuscript are available in the Supplementary Data 2.”

Human research participants

Policy information about studies involving human research participants and Sex and Gender in Research.

Reporting on sex and gender	All patients enrolled in the study were male (biological attribute).
Population characteristics	All-male, ranging between 56 till 91 years diagnosed with bladder cancer stage T1
Recruitment	All patients were informed about the study and provided a signed informed consent according with the Central Lisbon Hospital Center Central Scientific Committee. Selection of patients to enroll the study was based on the following criteria: (a) Inclusion: clear bladder cancer diagnosis, and (b) Exclusion: no records of urinary cancer history, no HIV, no organ transplant, and no recent chemo/radiotherapy
Ethics oversight	Central Lisbon Hospital Center Ethics Committee. The approval ethics committee number is 669/2018.

Note that full information on the approval of the study protocol must also be provided in the manuscript.

Field-specific reporting

Please select the one below that is the best fit for your research. If you are not sure, read the appropriate sections before making your selection.

☒ Life sciences ☐ Behavioural & social sciences ☐ Ecological, evolutionary & environmental sciences

For a reference copy of the document with all sections, see nature.com/documents/nr-reporting-summary-flat.pdf

Life sciences study design

All studies must disclose on these points even when the disclosure is negative.

Sample size	Sample size was chosen based on the avaiable data
Data exclusions	There were no data exclusions
Replication	Each urine sample were analysed with two technical replicates and with 2 LC-MS injections
Randomization	For each cohort, samples were randomized prior to mass spectrometry-based proteomics analysis
Blinding	Blinding was not applicable to our study design

Reporting for specific materials, systems and methods

We require information from authors about some types of materials, experimental systems and methods used in many studies. Here, indicate whether each material, system or method listed is relevant to your study. If you are not sure if a list item applies to your research, read the appropriate section before selecting a response.

Materials & experimental systems

n/a	Involved in the study	
☐	☒	Antibodies
☒	☐	Eukaryotic cell lines
☒	☐	Palaeontology and archaeology
☒	☐	Animals and other organisms
☒	☐	Clinical data
☒	☐	Dual use research of concern

Methods

n/a	Involved in the study	
☒	☐	ChIP-seq
☒	☐	Flow cytometry
☒	☐	MRI-based neuroimaging

Antibodies

Antibodies used	ab136943 - Myeloperoxidase (MPO) Human ELISA kit from abcam
Validation	ELISA MPO assay
